# Correction to “Divergent progression pathways in male androgenetic alopecia and female pattern hair loss: Trichoscopic perspectives”

**DOI:** 10.1111/jocd.16421

**Published:** 2024-06-14

Kamishima T, Hirabe C, Myint KZY, Taguchi J. Divergent progression pathways in male androgenetic alopecia and female pattern hair loss: Trichoscopic perspectives. *J Cosmet Dermatol*. 2024;23(5):1828‐1839. http://doi.org/10.1111/jocd.16177

In Table 2B of page 6, each set of rows was one row off. The corrected table is below.

**TABLE 2:** (B) Correlation between a‐Sinclair and quantitative trichoscopic factors in FPHL.

		All: a‐Sinclair I–V (*N* = 57)		NS: a‐Sinclair I–II (*N* = 20)		S: a‐Sinclair III–V (*N* = 37)	
		rs	*p*‐Value	rs	*p*‐Value	rs	*p*‐Value
Hair diameter	Max D	−0.31933	0.01547	0.03991	0.86363	−0.07052	0.68725
	VH%	0.6276	<0.00001	0.39728	0.07454	0.51989	0.00115
	IH%	0.30315	0.02189	0.14349	0.53493	0.14234	0.40785
	TH%	−0.63819	<0.00001	−0.27032	0.23596	−0.47978	0.00304
Hair‐number / FU	1FU%	0.544	1.00E−05	0.58797	0.00506	0.47205	0.00365
	2FU%	−0.27332	0.03967	−0.3815	0.0879	−0.25116	0.13953
	3FU%	−0.44277	0.00056	−0.46129	0.03531	−0.1872	0.27427
	MFU%	−0.29542	0.02568	−0.14307	0.53614	−0.33834	0.04355
Hair density	THC	−0.2766	0.03726	0.11942	0.60615	−0.1849	0.25466

*Note*: Green: Hair diameter‐related trichoscopic factors; blue: hair number per FU‐related trichoscopic factors; yellow: hair density‐related trichoscopic factors. Correlation coefficients (rs‐values) greater than |0.2| with statistical significance (p < 0.05) are denoted by colors.

Abbreviations: 1FU%, 2FU%, 3FU%, MFU%, single‐hair, double‐hair, triple‐hair, multiple‐hair per follicular unit rate; a‐Sinclair, adopted Sinclair scale; FPHL, female pattern hair loss; FU, follicular unit; IH%, indeterminate hair rate; Max D, maximum hair diameter; TH%, terminal hair rate; THC, total hair count; VH%, vellus hair rate.

We apologize for this error.

